# First Metabolic Insights into *Ex Vivo Cryptosporidium parvum*-Infected Bovine Small Intestinal Explants Studied under Physioxic Conditions

**DOI:** 10.3390/biology10100963

**Published:** 2021-09-26

**Authors:** Juan Vélez, Liliana M. R. Silva, Ulrich Gärtner, Arwid Daugschies, Sybille Mazurek, Carlos Hermosilla, Anja Taubert

**Affiliations:** 1 Institute of Parasitology, Biomedical Research Center Seltersberg, Justus Liebig University Giessen, Schubert Str. 81, 35392 Giessen, Germany; liliana.silva@vetmed.uni-giessen.de (L.M.R.S.); carlos.r.hermosilla@vetmed.uni-giessen.de (C.H.); anja.taubert@vetmed.uni-giessen.de (A.T.); 2 Institute of Veterinary Physiology and Biochemistry, Justus Liebig University Giessen, Frankfurter Str. 100, 35392 Giessen, Germany; sybille.mazurek@vetmed.uni-giessen.de; 3 Institute of Anatomy and Cell Biology, Justus Liebig University Giessen, Aulweg 123, 35392 Giessen, Germany; ulrich.gaertner@anatomie.med.uni-giessen.de; 4 Institute of Parasitology, University of Leipzig, An den Tierkliniken 35, 04103 Leipzig, Germany; daugschies@vetmed.uni-leipzig.de

**Keywords:** bovine small intestinal explants, *Cryptosporidium parvum*, glycolysis, glutaminolysis, metabolism, bovine small intestinal epithelial cells

## Abstract

**Simple Summary:**

As the most relevant zoonotic cause of cryptosporidiosis, *C. parvum* infects cattle worldwide. In vitro studies on *C. parvum* are absent on the most important animal host under physiological oxygen conditions of the intestine. The aim of this study was to rectify this lack of knowledge, and to deliver a practical model to study *C. parvum*–host cell–intestinal microbiome interactions in the metabolic context. The present metabolic analyses of *C. parvum*-infected bovine small intestinal (BSI)-explants revealed a parasite-dependent reduction in important metabolic activities (e.g., glycolysis, glutaminolysis) at 3 hpi (hours post-infection) followed by striking increases in the same metabolic functions at 6 hpi, thus paralleling previously reported metabolic impacts of *C. parvum* on humans. In addition, PCA analysis confirmed physiological oxygen concentrations as a driving factor of metabolic responses in infected BSI explants. The present model allows the study of *C. parvum*-triggered metabolic modulation of intestinal cells. Moreover, this realistic platform offers the possibility to address pending questions regarding *C. parvum*–host cell–intestinal microbiome interactions. Thus, the present approach may deliver important insights into how to promote the innate immune system–intestinal microbiome alliances, which maintain the epithelial integrity of the gut thereby supporting human and animal health.

**Abstract:**

The apicomplexan *Cryptosporidium parvum* causes thousands of human deaths yearly. Since bovines represent the most important reservoir of *C. parvum*, the analysis of infected bovine small intestinal (BSI) explants cultured under physioxia offers a realistic model to study *C. parvum*–host cell–microbiome interactions. Here, *C. parvum*-infected BSI explants and primary bovine small intestinal epithelial cells were analysed for parasite development and metabolic reactions. Metabolic conversion rates in supernatants of BSI explants were measured after infection, documenting an immediate parasite-driven metabolic interference. Given that oxygen concentrations affect cellular metabolism, measurements were performed at both 5% O_2_ (physiological intestinal conditions) and 21% O_2_ (commonly used, hyperoxic lab conditions). Overall, analyses of *C. parvum*-infected BSI explants revealed a downregulation of conversion rates of key metabolites—such as glucose, lactate, pyruvate, alanine, and aspartate—at 3 hpi, followed by a rapid increase in the same conversion rates at 6 hpi. Moreover, PCA revealed physioxia as a driving factor of metabolic responses in *C. parvum*-infected BSI explants. Overall, the *ex vivo* model described here may allow scientists to address pending questions as to how host cell–microbiome alliances influence intestinal epithelial integrity and support the development of protective intestinal immune reactions against *C. parvum* infections in a realistic scenario under physioxic conditions.

## 1. Introduction

The apicomplexan parasite *Cryptosporidium parvum* affects both humans and animals worldwide [1,2,3,4,5]. This water- and food-borne parasite, together with other diarrheal diseases, was identified in 2010 as causing a higher global mortality than AIDS, malaria, and measles together [2,5]. Currently, there are only two approved drugs against cryptosporidiosis; both nitazoxanide for humans [6] and halofuginone lactate for bovines [4,7]—lack good efficacy against *C. parvum*. Thus, especially in susceptible hosts—such as infants, immunocompromised individuals, and newborn calves—anti-cryptosporidial treatments do little to improve health conditions [4,6,7].

Based on genome sequencing [8], knowledge of *C. parvum*-related survival strategies has increased tremendously in recent years, and has led to the identification of general metabolic patterns—especially with respect to glycolysis as a key source of energy supply [9,10,11,12,13]. Nevertheless, host species-related differences in the *C. parvum*-driven metabolic impact on host cells have also been demonstrated [10,14,15]. Previous *in vivo* studies evidenced the importance of the microbiotic composition in the outcome of cryptosporidiosis, i.e., either by delaying the onset of oocyst shedding, or by reducing the total number of shed oocysts, in murine and ruminant models, respectively [4,16]. Strikingly, the intracellular signal molecule indole—which is produced by almost 85 different bacteria [17,18], and is related to the inhibition of biofilm formation [19,20]—was also identified as biomarker of human cryptosporidiosis outcomes [17].

The complex host- and microbiome-dependent differences in *C. parvum*-related virulence evidence the necessity of expanding scientific efforts beyond *in vitro* cell culture systems under hyperoxic conditions (21% O_2_) and immunosuppressed murine models, which reflect neither *in vivo* intestinal physioxia nor host innate immune reactions of humans and ruminants against *C. parvum* [13,21]. Similarly, it also seems relevant to include the most important zoonotic reservoir species, i.e., domestic bovine species [13,22]. As pointed out elsewhere, cryptosporidiosis is not only a neglected anthropozoonotic disease, but also of high concern in terms of the One Health concept [23,24]. Therefore, a better understanding of metabolic parasite–host cell interactions in bovines may provide valuable insights for the adequate control of cryptosporidiosis in both humans and bovines. To date, information on metabolic alterations/changes in *C. parvum*-infected cattle is scarce, probably due to both high animal maintenance costs and limited availability of species-specific metabolic tools [25]. To improve current knowledge on *C. parvum*-driven metabolic changes in bovine small intestinal epithelial cells, studies considering physiological oxygen conditions and primary host cell types are urgently needed [13]. In this sense, individual bovine small intestinal (BSI) explants, which include the individual microbiome of the host, may provide a useful and uncostly model for *ex vivo* analysis of *C. parvum*–host epithelial cell–microbial consortia interactions. Considering all of this, we intended herein to refocus cryptosporidiosis-related experimentation by including host- and *C. parvum*-specific microenvironmental parameters, working for the first time with BSI explants and primary bovine small intestinal epithelial cells (BSIEC) under physiological oxygen conditions to better mimic the *in vivo* situation.

## 2. Materials and Methods

### 2.1. Isolation of Bovine Small Intestinal (BSI) Explants

BSI explants were isolated from crossbreed beef cattle (Fleckvieh) (approximately 2 years old, *n* = 3) from a local slaughterhouse. A 6 cm long duodenum segment was separated from the small intestine using a pair of sterile tweezers and scissors. Isolated small intestinal segments were immediately transferred to 500 mL glass flasks containing sterile 1X PBS supplemented with 500 U/mL of penicillin, 500 ug/mL of streptomycin, and 12.5 µg/mL of amphotericin B (1X antibiotic–antimycotic solution, Gibco, Grand Island, NY, USA), and transported immediately on ice (4 °C) to the Institute of Parasitology, Justus Liebig University Giessen, Germany. For isolation of BSI explants, the tunica serosa and adipose tissue of intestinal segments were separated from the external tunica muscularis using a sterile scalpel, after which they were longitudinally opened under sterile laminar flow conditions and washed thrice with cold 1X PBS containing 1X antibiotic–antimycotic solution (antibiotic–antimycotic solution (100X), Gibco, Grand Island, NY, USA). BSI explants of 8 mm^2^ (*n* = 24, 3 BSI explants for each condition) were obtained via sterile biopsy punches (pfm medical, Solingen, Germany) and individually placed mucosa-upwards onto biopsy foam pads (Fisher Scientific, Schwerte, Germany) in 6-well cell culture plates (Merck, Darmstadt, Germany) [26]. Complete Dulbecco’s modified Eagle’s medium/nutrient mixture F-12 (DMEM/F-12, Gibco) cell culture medium containing 10% foetal bovine serum (FBS; Biochrom AG, Berlin, Germany), 100 UI penicillin, and 0.1 mg streptomycin/mL (both Merck)—and additionally supplemented with recombinant murine noggin (10 ng/mL, PeproTech, Hamburg, Germany), osteoprotegerin (100 ng/mL, OPG, Merck), and human EGF recombinant protein (25 ng/mL, EGF, Gibco)—was used for BSI explant cultures, as previously described [27].

### 2.2. Isolation of Bovine Small Intestinal Epithelial Cells (BSIEC) from Intestinal Crypts

Bovine small intestinal crypts were isolated as described by Ren et al. (2017), with slight modifications [28]. Briefly, intestinal samples were washed with cold 1X PBS containing 1X antibiotic–antimycotic solution (Gibco), and the tunica mucosa was isolated using a sterile scalpel, thereby avoiding touching the tunica muscularis. Mucosal tissue was cut into small pieces using sterile scissors, transferred into 50 mL Falcon tubes, and washed twice in cold 1X PBS containing 1X antibiotic–antimycotic solutions (Gibco) as described above. Afterwards, samples were treated for 6 min (37 °C under agitation) with type I collagenase (200 U/mL, Merck) and hyaluronidase (100 U/mL, Stemcell, Cologne, Germany) in a tissue:enzyme ratio of 1:5. Thereafter, the tissue sample was dissociated by pipetting, and the supernatant was transferred to an equal volume of sterile FBS to inhibit enzyme activities. The cell solution was centrifuged (100× *g*, 5 min, 4 °C), the pellet was resuspended in the same growth medium used for *ex vivo* culture of BSI explants (see Section 2.1) and then filtered (100 μm pore size; Sarstedt, Nümbrecht, Germany). Individual crypts were counted microscopically and seeded at a density of 200 crypts/cm^2^ in sterile 25 cm^2^ plastic tissue culture plates (Gibco). The cell culture medium was changed after 24 h of incubation (21/5% O_2_; 5% CO_2_; 37 °C; ~70% relative humidity), and every second day thereafter.

### 2.3. BSI Explant- and BSIEC-Based Host Cell Culture Systems

Both BSI explants and BSIEC were cultured under two different oxygen atmospheres—namely at 5% (physioxia) and 21% O_2_ (hyperoxia)—to mimic both the physioxic conditions of the small intestine *in vivo* [13,29], and hyperoxic conditions as typically applied in the lab conditions used in most studies on *C. parvum*-infected host cells *in vitro* [13,30].

Physioxic conditions (5% O_2_) were achieved in a physiological atmosphere working chamber (InvivO_2_^®^ 400, Ruskinn, Vienna, Austria), in parallel to the hyperoxic culture conditions (21% O_2_) maintained in a standard cell culture incubator (Heracell 240i, Thermo Scientific, Langenselbold, Germany). Cell media for physioxic cultures were equilibrated with oxygen by placing them on a rocker for half a day within the working chamber (InvivO_2_ 400) [13,29,30]. In the case of BSI explants, cultures were maintained for up to 3 and 6 h, and for BSIEC up to 24 and 48 h. For detailed primary epithelial cell characterization as well as cell vitality assessment, BSIEC monolayers were controlled for up to 4 weeks after isolation.

### 2.4. Characterization of BSIEC

To confirm the epithelial nature of crypt-derived BSIEC, monoclonal anti-pan-cytokeratin antibodies (C2562, FITC conjugate, Merck) were used according to previously published protocols [28,31]. Moreover, fibroblast contamination or eventual mesenchymal transformation during cell division/growth was controlled via type III intermediate filament detection, using a monoclonal anti-vimentin-Cy3 antibody (C9080, Merck), as described elsewhere [28]. In addition, cryosections of bovine small intestines were used as antibody controls for both epithelial and mesenchymal cells. Briefly, crypt-derived BSIEC from six different donors (*n* = 6) were fixed in triplicate with paraformaldehyde (4%, 15 min, room temperature (RT), Merck) and then blocked/permeabilized in 3% bovine serum albumin (BSA; Thermo Fisher Scientific, Waltham, MA, USA) and 0.3% Triton X-100 (block/permeabilization; Sigma-Aldrich, Taufkirchen, Deutschland) solution for 15 min. Samples were probed with primary anti-cytokeratin antibodies (C2562, Sigma-Aldrich, 1:100) for 60 min at RT, followed by incubation in secondary antibody solutions (F9137, Sigma-Aldrich, 1:100, 30 min, in the dark at RT). Cell layers were then washed thrice in 1X PBS and probed with monoclonal anti-vimentin cy3-conjugated clone V9 (C9080, Merck, 1:100; RT, in the dark). Afterwards, samples were mounted in DAPI-containing anti-fading buffer (Fluoromount G with DAPI; Thermo Fisher Scientific, Waltham, MA, USA). Representative images were taken using an epifluorescence microscope (Olympus IX81, Hamburg, Germany) equipped with a digital camera (Olympus XM10, Hamburg, Germany).

### 2.5. Parasites

*C. parvum* oocysts of subtype 60-kDa glycoprotein (gp60) IIaA15G2RI, were kindly provided by the Institute of Parasitology, Faculty of Veterinary Medicine, University of Leipzig, Germany, and originated from experimentally infected calves [13,25]. For storage of *C. parvum* oocyst stocks, phosphate-buffered saline (PBS, pH 7.4) containing 100 UI penicillin and 0.1 mg streptomycin/mL (Merck) was used and replaced monthly for up to three months in order to maintain sporozoite infectivity as described in [13,32].

### 2.6. BSI Explant and BSIEC Infections with Cryptosporidium parvum Sporozoites

For sporozoite isolation, *C. parvum* oocysts were excysted using the protocol of Varughese et al. [33], with minor modifications. Briefly, oocysts were pelleted (5000× *g* for 5 min, 4 °C), resuspended in acidified (pH 2.0), prewarmed (37 °C) 1X Hank’s Balanced Salt Solution (HBSS; Merck), and incubated for 10 min at 37 °C. Then, a second centrifugation step was performed (5000× *g* for 5 min, 4 °C), followed by a second incubation in non-acidified 1X HBSS for 10 min at 37 °C. Afterwards, freshly excysted sporozoites were pelleted and resuspended in cell culture medium (Section 2.1). For host cell infections, excysted oocysts were added to BSI explants (2.5 × 10^5^ oocysts/BSI explant) and to BSIEC monolayers using a multiplicity of infection (MOI) of 0.5:1.

### 2.7. Scanning Electron Microscopic Analysis of Cryptosporidium parvum-Infected BSI Explants

Scanning electron microscopy (SEM)-based analysis was conducted on *C. parvum*-infected and non-infected (controls) BSI explants fixed in 2.5% glutaraldehyde (Merck) and post-fixed in 1% osmium tetroxide (Merck). Afterwards, samples were washed in distilled water, dried by critical point CO_2_-treatment, and sputtered with gold particles [13]. Samples were analysed using a Philips XL30 scanning electron microscope at the Institute of Anatomy and Cell Biology, Justus Liebig University Giessen, Germany, as described elsewhere [13].

### 2.8. Immunofluorescence-Based Quantification of Cryptosporidium parvum Infection Rates in BSIEC

For infection rate determination in BSIEC, a *Vicia villosa* lectin (VVL; Vector Laboratories, Burlingame, CA, USA)-based staining was applied as described elsewhere [12,30]. Briefly, BSIECs were washed in sterile 1X PBS once, fixed in 4% paraformaldehyde (Merck, 15 min), and again washed in 1X PBS. BSIEC were then treated with 0.3% Triton X-100 (Merck) and 3% BSA (Merck) for 1 h at RT. Then, *C. parvum* stages were labelled by VVL (1:2000 dilution, 45 min, RT, dark chamber). Afterwards, samples were mounted in Fluoromount-G™ Medium, with DAPI (Thermo Fisher Scientific, Waltham, MA, USA). Samples were analysed using an epifluorescence microscope (IX81 Olympus, 40× magnification). Images were taken for identification of parasitic stages—such as intracellular sporozoites, trophozoites, and meronts—and infection rates were estimated using ImageJ [13].

### 2.9. Tissue DNA Extraction

Both *C. parvum*-infected and non-infected BSI explants (*n* = 9, triplicates for each donor), as well as infection doses of sporozoites (internal control, *n* = 2.5 × 10^5^ oocysts per aliquot), were deposited into microtubes containing 2.8 mm diameter ceramic beads (Bertin Pharma, Hamburg, Germany) and submitted to 9 cycles of disruption (each cycle for 20 s, at 6 m/s, with a 10 s pause between cycles) using a bead disruptor (OMNI International, Kennesaw, GA, USA). Afterwards, the samples were processed for genomic DNA isolation using the QIAamp^®^ mini kit for tissue (Qiagen, Hilde, Germany), following the manufacturer’s instructions.

### 2.10. qPCR-Based Quantification of Cryptosporidium parvum Replication in BSI Explants

Quantitative qPCR-based amplification of *C. parvum*-specific heat shock protein 70 (hsp70) was performed as previously reported [25], with slightly modifications. Briefly, forward primer (CP_hsp70_fwd 5′-aactttagctccagttgagaaagtactc-3′), reverse primer (CP_hsp70_rvs 5′-catggctctttaccgttaaagaattcc-3′), and TaqMan probes (HSP_70_SNA 5′-aatacgtgtagaaccaccaaccaatacaacatc-3′, labelled at the 5′end with the reporter dye FAM (6-carboxyfluorescein) and at the 3′ end with the quencher dye TAMRA (6-carboxytetramethyl-rhodamine)) were purchased from Biomers (Ulm, Germany). The final qPCR reaction volume was 20 μL, containing 1X Polymerase (PerfeCTa FastMix II^®^, Quantabio, Beverly, MA, USA), 5 μL DNA template, 0.8 μL of forward and reverse primer (400 nM), 0.4 μL TaqMan probe (200 nM), and 0.2 μL BSA (10 mg/mL). qPCR reactions were performed in triplicate in a Rotor-Gene Q thermocycler^®^ (QIAGEN, Hilden, Germany) under the following conditions: 95 °C for 5 min, 45 cycles at 94 °C for 15 s, and 60 °C for 60 s. Negative controls consisting of gDNA from non-infected BSI explants, sporozoites (infection doses), and a plasmid standard of target sample template DNA were included in each reaction. 

### 2.11. Quantification of Metabolic Conversion Rates of Cryptosporidium parvum-Infected BSI Explant Supernatants

Metabolic conversion rates of *C. parvum*-infected and non-infected (negative controls) BSI explants (*n* = 18; three biological replicates and three technical replicates for each experimental condition) cultured under physioxia (5% O_2_) and hyperoxia (21% O_2_) were calculated as described elsewhere [13,34]. Briefly, individual BSI-explant-derived supernatants (1 mL medium/well and explant [8-mm^2^-size]) and technical medium replicates co-cultured in order to back-calculate production or consumption of individual metabolites were collected after 3 and 6 hpi, centrifuged (400× *g*, 10 min, 4 °C), aliquoted, frozen, and stored at −80 °C. For analysis, the supernatants were incubated at 95 °C for 15 min in order to inactivate enzyme activities within the FBS-containing medium and centrifuged at 8000× *g* for 10 min. The metabolite concentrations in the medium supernatants were determined photometrically using a respons^®^920 bench-top clinical analyser (DiaSys Deutschland Vertriebs GmbH, Flacht, Germany) on the basis of the NAD(P)H—NAD(P)+ redox system, by measuring the increase or decrease in the absorbance at 340 nm. In detail, the measurements were based on the following enzymatic reactions—glucose: hexokinase coupled with glucose 6-P dehydrogenase; pyruvate and lactate: lactate dehydrogenase; glutamate: glutamate dehydrogenase; glutamine: glutaminase coupled with glutamate dehydrogenase; alanine: glutamate pyruvate transaminase coupled with lactate dehydrogenase; serine: periodate coupled with lactate dehydrogenase; aspartate: glutamate oxaloacetate transaminase coupled with malate dehydrogenase [13,35,36]. Metabolic conversion rates were calculated in (nmol/(h* intestinal cells)) after normalization based on a standard curve of known concentrations of bovine intestinal cells, and using GAPDH/1 primers as previously described [37]. In addition, metabolic conversion rates were normalized to control medium samples without BSI explants, which were co-incubated in parallel to culture plates with BSI explants.

### 2.12. Analysis of Glycolytic Responses of Cryptosporidium parvum-Infected BSIEC via Seahorse Technology

*C. parvum*-induced glycolytic responses in BSIEC were evaluated via the Glycolysis Stress Test Kit^®^ (Agilent, Ratingen, Germany), using a Seahorse XFp^®^ extracellular flux analyser (Agilent, Ratingen, Germany) as previously reported [13]. For each assay, 10^3^ BSIEC were plated in triplicate into XFp cell microplates (Agilent) for each experimental condition (infected and non-infected), allowed to grow for 24 h (~80% of cell confluence), and were then infected with *C. parvum* sporozoites, applying a parasite:host cell ratio of 0.5:1. Evaluation of mitochondrial oxygen consumption rates (OCR) and extracellular acidification rates (ECAR) was conducted on cells cultured in parallel at 3, 6, and 9 hpi, following the manufacturer’s protocol for the Glycolysis Stress Test Kit^®^. In brief, hydrated Seahorse XF 8-well plastic cartridges (Agilent) were incubated in a CO_2_-free incubator (Thermo Fischer Scientific, Waltham, MA, USA; 37 °C, 45 min). Then, the cell culture medium in all XFp cell microplate wells was replaced by DMEM-based medium (Agilent, Ratingen, Germany) containing 2 mM glutamine (Merck). Afterwards, glucose (10 mM), oligomycin (2.9 mM), and 2-DG (60 mM) (supplied by the kit) were sequentially supplemented to each cell sample via instrument-own injection ports (following 3 cycles of mixing and measurements). After measurements, *C. parvum*-infected and non-infected BSIEC were fixed by adding 4% paraformaldehyde (Merck) in 1X PBS and stored at 4 °C for subsequent cell and parasite counting (VVL-based immunofluorescence assay, see Section 2.7) for quantification of infection rates and normalization of flux rates.

### 2.13. Data Processing and Statistical Analysis

Overall, significances were determined by *p*-values < 0.05 in both *C. parvum* replication analyses and on glycolytic function tests by applying *t*-tests. For evaluation of significant reactions in metabolic conversion rates, the Kruskal–Wallis test was performed, followed by Dunn’s multiple comparison test. The methods used for clustering purposes were heatmaps and principal component analyses (PCAs), which were applied to metabolic conversion rates of metabolites after normalization by transforming raw data into percentages (setting the highest measurement of metabolic conversion rate found among the dataset to 100%). Concerning glycolytic activity measurements using Seahorse XFp^®^ technology, data were plotted as bar graphs, points, and connecting lines as well as scatterplots (presenting mean ± SD). Uppercase Ns represent the number of biological replicates, while lowercase ns represent technical replicates. Significances and data were plotted using Graph Pad^®^ v. 9.00 software (San Diego, CA, USA). PCA of metabolic conversion rates of *C. parvum*-infected and non-infected BSI explants under both physioxia and hyperoxia was performed using RStudio^®^ version 1.4.1103 [38] with R version 4.0.3 (2020-10-10) [39]. Briefly, the dataset of metabolic conversion rates was exported as an *.xlsx file, then imported into R using the xlsx^®^ package [40]. PCA was then performed on this dataset, followed by different transformations, as described in the script provided in the Supplementary Materials.

## 3. Results

### 3.1. Cryptosporidium parvum Replication in BSI Explants under Physioxic Oxygen Conditions

**Figure 1:**
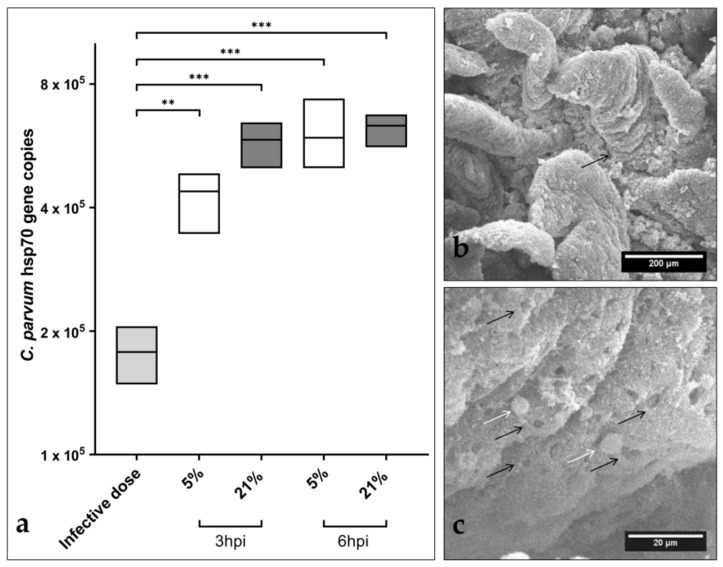
Early *Cryptosporidium parvum* development in bovine small intestinal (BSI) explants under both physioxic (5% O_2_) and hyperoxic (21% O_2_) conditions. A significant increase in parasite replication was detected in BSI explants (*n* = 3) via *C. parvum* hsp 70 gene-specific qPCR analyses, and by comparing 3 and 6 hpi with the initial sporozoite infection dose (i.d.), boxplots represent mean ± SD (**a**). SEM analysis of BSI explants confirmed parasite replication by revealing *C. parvum*-infected villi (black arrow) (**b**). Interestingly, *C. parvum*-infected BSI explants presented typical *C. parvum*-induced hole-like lesions in epithelial cells (black arrows), along with development of trophozoite-like stages (white arrows), which were detected as early as 3 hpi (**c**). Statistical significance (** *p* < 0.01, *** *p* < 0.001) was determined by Kruskal–Wallis test followed by Dunn’s multiple comparison test comparing infected BSI explants with initial sporozoite numbers used for infection (infection doses, *n* = 3). qPCR- and SEM-based analyses were performed in duplicate and triplicate, respectively.

*C. parvum* hsp70-based qPCR revealed a significant increase in parasite numbers in infected BSI explants after 3 and 6 hpi under both oxygen conditions, when compared to hsp70-gene copies from infection doses used for BSI explant infection (Figure 1). However, considering different infection times (3 and 6 hpi) or different oxygen conditions, significant differences in parasite numbers were not observed (Figure 1). Moreover, evaluation of parasite replication at later timepoints (24 and 48 hpi) revealed a decrease in parasite gene copies (see Figure S1), indicating that the parasite failed to continuously proliferate. SEM-based analysis of *C. parvum*-infected BSI explants evidenced structural integrity of villi at 3 and 6 hpi (Figure 1a). Moreover, microscopic estimation of parasite numbers corresponded well to qPCR-based quantification of parasite gene copies, suggesting that *C. parvum* was indeed able to infect and replicate in BSI explants, which comprised all important cell types of typical *C. parvum*-infected small intestinal villi *in vivo*, i.e., intestinal stem cells, Paneth cells, tuft cells, enteroendocrine cells, and goblet cells, in addition to enterocytes—the latter representing typical host cells for *C. parvum* intracellular replication. SEM analysis confirmed intracellular *C. parvum* stages in BSI explants from 3 hpi onwards, thereby illustrating trophozoite and/or meront stages (Figure 1b). Interestingly, both SEM- and hematoxylin and eosin (H&E) staining-based analyses performed at later timepoints of infection evidenced progressive degradation of BSI explants from 12 hpi onwards (data non-shown), which may have hampered continuous parasite development, as also reflected by qPCR (Figure S1).

The current experimental approach also intended to maintain each BSI explant and its individual microbiome under conditions reflecting physioxic oxygen conditions of 5% O_2_ (Note: 1–10% O_2_ is commonly found in the small intestinal lumen of mammalian hosts [29]). As an interesting finding, SEM analyses showed parasite-driven hole-like lesions in the epithelial surfaces of parasitized villi, as reported for both *in vitro* experiments with permanent host cells [13] and *in vivo* lesions in *C. parvum*-infected bovine small intestines [41,42].

Overall, the present findings on increased parasite gene copies early after infection (Figure 1a), on the presence of intracellular parasitic stages (Figure 1c, white arrows), and on typical parasite-driven host-cell membrane damage (Figure 1c, black arrows) strongly indicate that *C. parvum* sporozoites indeed infected epithelial cells, transformed into trophozoites, and further developed into their first merogonic stages, thereby suggesting BSI explants as a suitable system for the early timeframe of parasite replication (time window: up to 6 hpi) to study *C. parvum*-mediated host cell recognition, sporozoite invasion, transformation into trophozoites, and early merogony.

**Figure 2:**
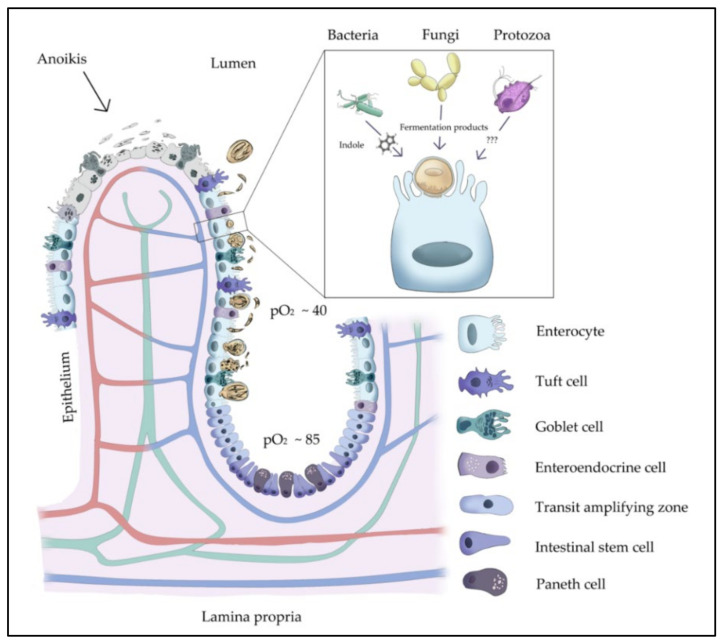
Illustration of possible microbiota–host epithelial cell–*C. parvum* interactions, with description of diverse specialized gut cells of the small intestinal villi. It has been shown that bacteria-derived indole might act as a faecal-derived biomarker for cryptosporidiosis susceptibility in humans. In addition, the faecal-bacteria-derived indole was suggested to define the metabolic interaction between bacteria and *C. parvum*. On the other hand, yeast-derived molecules have been proven to reduce infection-derived pathological lesions in bovines and, thus, used to ameliorate cryptosporidiosis in calves in the field. pO_2_ is presented as mm/HG.

This *ex vivo* model allows not only the inclusion of all specialized cell types in the intestinal niche (Figure 2), but also individual intestinal micro- and mycobiomes, which have been demonstrated to influence cryptosporidiosis outcomes in both humans and bovines [4,17] (Figure 2). Bacteria- and yeast-derived molecules (i.e., bacterial indole and yeast-derived fermentation products) have been revealed as molecular markers of cryptosporidiosis in humans [17] and proven to reduce enteritis-derived symptoms of naturally *C. parvum*-infected-calves in the field [4,43], respectively.

### 3.2. Metabolic Signatures of Cryptosporidium parvum-Infected BSI Explants Depend on Oxygen Concentrations

**Figure 3:**
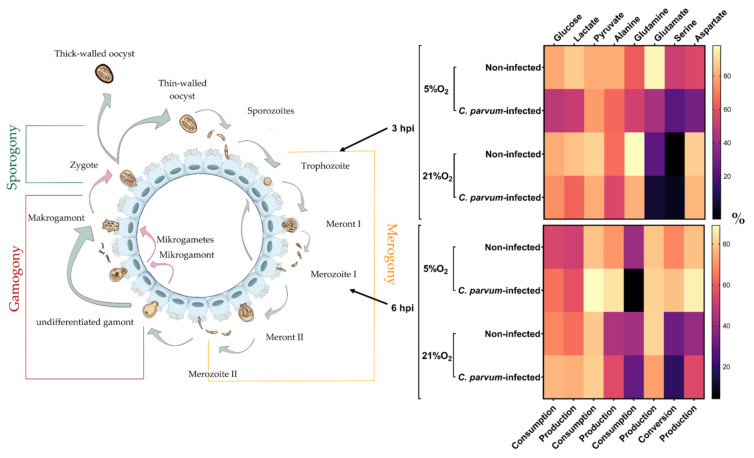
Conversion rates of key metabolites (glucose, lactate, pyruvate, alanine, glutamine, glutamate, serine, and aspartate) in supernatants of *C. parvum*-infected and non-infected bovine small intestinal (BSI) explants within the early phase of the parasitic life cycle. Metabolite conversion rates were analysed at 3 and 6 hpi under physioxic (5% O_2_) and hyperoxic (21% O_2_) conditions. Heatmaps present metabolic conversion rates as the mean (*n* = 3) of normalized percentages (legend on the right) across the two analysed oxygen concentrations and timepoints post-infection. Serine conversion represents consumption at 5% O_2_ 3 hpi and 6 hpi, while at 21% O_2_ 3 hpi and 6 hpi serine was also partially produced. Metabolic conversion rates scale from low (dark) to high (bright).

**Figure 4:**
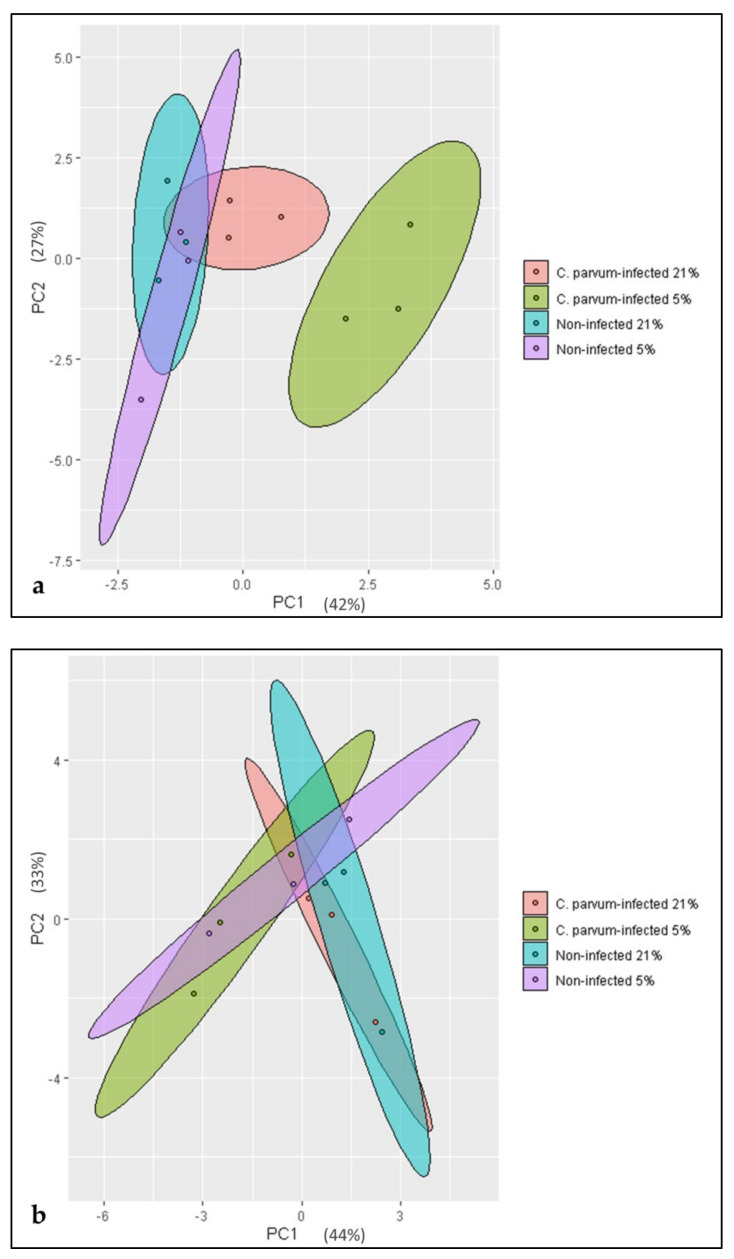
Principal component analysis (PCA) of metabolic data of *Cryptosporidium parvum*-infected and non-infected bovine small intestinal (BSI) explants under physioxic (5% O_2_) and hyperoxic (21% O_2_) conditions at 3 hpi (**a**) and 6 hpi (**b**). The metabolic impact exerted by infection under physioxia was shown to be the major factor contributing to the variability of the dataset at 3 hpi (**a**). In contrast, at 6 hpi, the hyperoxic conditions—independent of infection—tend to explain a greater extent of the variability of metabolic conversion rates (**b**).

Metabolic conversion rates were analysed in supernatants of *C. parvum*-infected and non-infected BSI explants propagated under both physioxic (5% O_2_) and hyperoxic (21% O_2_) conditions. Three hours after infection, glucose consumption, lactate production, pyruvate consumption, alanine production, glutamine consumption, glutamate production, and serine and aspartate production decreased in infected BSI explants in an oxygen-independent manner, even though a more pronounced effect was observed under physioxic conditions (Figure 3, 3 hpi). Serine conversion shifted from decreased consumption in infected BSI explants cultivated in the presence of 5% O_2_ to serine production when the explants were cultivated at 21% O_2_. In addition, a higher impact under physioxia was also reflected by higher PC1 values of a PCA, which explained more than 60% of the variability in the total dataset (Figure 4a). Considering oxygen conditions, serine conversion differed markedly, since consumption of this metabolite was observed at 5%, whilst it was partially produced at 21% O_2_.

Only 3 h later (= 6 hpi), the metabolic situation reversed entirely in *C. parvum*-infected BSI explants. Thus, glucose consumption, lactate production, pyruvate consumption, alanine production, and aspartate production increased in infected cells independent of the oxygen supply. Glutamate production and serine consumption decreased in infected BSI explants at 21% O_2_, but increased under physioxic conditions, thereby following the general trend towards increased conversion rates at 6 hpi. Glutamine consumption was the only value that decreased in infected BSI explants at 6 hpi regardless of the oxygen supply.

The metabolic data were subjected to principal component analysis (PCA), which revealed a time- and infection/oxygen-dependent variability of the observed metabolic conversion rates. Thus, at 3 hpi an infection-driven clustering of samples into the four experimental groups (Figure 4a) was present. Here, the influence of *C. parvum* infection seemed to be of major importance and caused a higher variability in the dataset. Thus, *C. parvum*-infected BSI explants under both O_2_ conditions—but especially under physioxia—presented higher PC1 values when compared to non-infected groups. At 6 hpi, this constellation changed, and PCA revealed a major influence of oxygen conditions (Figure 4b). Thus, cells cultivated under hyperoxic conditions were displaced towards the PC1 field irrespective of *C. parvum* infection. Thus, hyperoxia contributed to the enhanced variability of data observed at 6 hpi, and probably corresponded to the documented slowdown of intracellular parasite replication.

### 3.3. Characterization and Cryptosporidium parvum-Driven Reactions in Primary BSIEC

**Figure 5:**
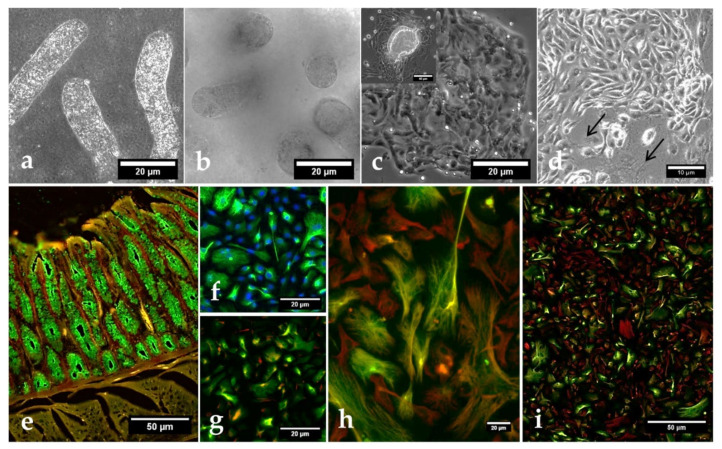
Morphology and characterization of bovine small intestinal epithelial cells (BSIEC). Villi (**a**) and crypts (**b**) after collagenase–hyaluronidase digestion, cell aggregates originating from attached crypts at 24 h post-isolation (c upper left photo), and well-differentiated BSIEC clusters with cobblestone cell morphology at 48 h post-isolation (**c**). Mixture of epithelial cells and fibroblasts (arrows) observed at 4 days post-isolation (**d**). For cell-type characterization, BSIEC were stained with cytokeratin (green, marker of epithelial cells), vimentin (red, marker of mesenchymal cells) and, eventually, with DAPI (blue, DNA). Cryosection of bovine small intestine (positive control) (**e**). (**f**–**i**): BSIEC after one week (**f**), two weeks (**g**), and three weeks (**h**–**i**) of *in vitro* culture.

To explore a further infection system being rather close to the *in vivo* situation, we additionally isolated primary BSIEC, which represent the natural host cells of *C. parvum* within the intestinal niche. Using a collagenase I/hyaluronidase-based protocol [28], villi (Figure 5a) and proliferative single crypts (Figure 5b) were isolated and cultured into confluent epithelial cell layers within 4 days. As expected, proliferative BSIEC clusters (Figure 5c) maintained new generations of epithelial cells with typical cobblestone morphology over several days (Figure 5c,e). Interestingly, from 4 days post-isolation onwards, enhanced contamination with fibroblasts was observed in BSIEC cultures, thereby leading to a mixed population of fibroblasts and epithelial cells within BSIEC monolayers (Figure 5e).

BSIEC isolates (*n* = 6) were characterized by both cytokeratin (= marker of epithelial cells) and vimentin (= marker of mesenchymal cells) staining. In addition, cryosections of bovine small intestines were used for positive controls, and presented a cytokeratin-positive epithelial brush border and vimentin-positive subepithelial cells (Figure 5e). As expected, BSIEC stained positive with cytokeratin, but showed no reactions with vimentin early after isolation (Figure 5g–i), thereby illustrating their epithelial origin. As reported above, with ongoing culture duration, fibroblasts (=vimentin-positive) emerged, and led to mixed cultures from 4 days onwards (Figure 5h,i). Consequently, only freshly isolated BSIEC from up to 2 days of culture were used in the current *C. parvum*-related experiments.

**Figure 6:**
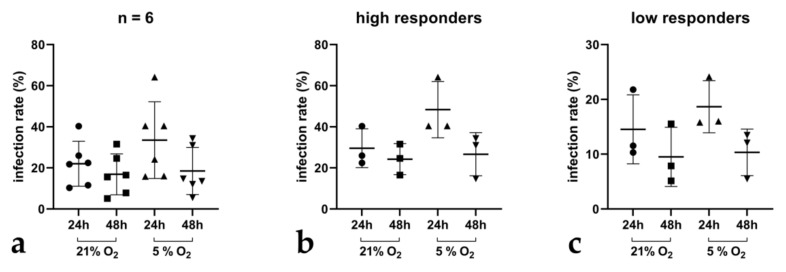
*Cryptosporidium parvum* development in BSIEC under physioxic and hyperoxic conditions. In total, 6 individual BSIEC isolates were generated and thereafter infected with freshly excysted *C. parvum* sporozoites (**a**). Within these populations, high (**b**) and low (**c**) infected BSIEC isolates were identified.

To test the suitability of BSIEC as host cells for *C. parvum*, BSIEC isolates (*n* = 6) were infected and monitored for intracellular parasite development via VVL-based immunofluorescence for up to 48 h under physioxic and hyperoxic conditions. Overall, a drop in infection rates was revealed from 24 to 48 h after infection, showing that BSIEC supported parasite infection but not replication under the used conditions (Figure 6a). Overall, infection rates varied in a donor-dependent manner; thus, individual BSIEC isolates (*n* = 6) were separated into high and low responders for *C. parvum* infections (Figure 6b,c). Mean infection rates ranged from 33.5% ± 50.5 at 24 hpi to 18.5% ± 13.5 at 48 hpi under physioxic conditions, and from 28% ± 21 at 24 hpi to 21.29% ± 14 at 48 hpi under hyperoxic conditions. Thus, by tendency, *C. parvum* infection rates in BSIEC monolayers seemed slightly lower under physioxia (5% O_2_).

**Figure 7:**
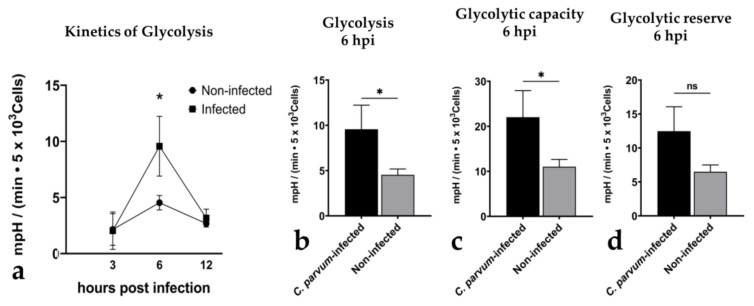
Glycolytic responses in *Cryptosporidium parvum*-infected BSIEC. Glycolytic responses were monitored via extracellular acidification rates (ECAR) in *C. parvum*-infected and non-infected BSIEC at 3, 6, and 12 hpi, evidencing a parasite-driven upregulation of glycolysis (**a**,**b**) and glycolytic capacity (**c**) at 6 hpi. An increase in glycolytic reserve was also observed (**d**), but proved barely significant. Statistical significance (* *p* < 0.05, ns = non-significant) was determined via *t*-test, comparing infected to non-infected (controls), followed by Dunnett’s test correction. Bars represent mean ± SD (*n* = 3).

To finally analyse infection-driven reactions in BSIEC, they were tested for metabolic responses at 3, 6, and 12 hpi by estimating oxygen consumption rates (OCR) and extracellular acidification rates (ECAR) via Seahorse^®^ technology (Figure S2). When referring to kinetics, a significant increase in ECAR was detected at 6 hpi (infected BSIEC vs. controls: *p* = 0.03, Figure 7a), thereby paralleling data on infected BSI explants (see Figure 3, 6 hpi). When deciphering this timepoint (6 hpi) in more detail, a significant infection-driven enhancement of total glycolysis (infected BSIEC vs. controls: *p* = 0.03, Figure 7b) and glycolytic capacities (infected BSIEC vs. controls: *p* = 0.03, Figure 7c) was calculated. Moreover, the glycolytic reserve was also increased in *C. parvum*-infected BSIEC (Figure 7d), even though statistics did not show significance (infected BSIEC vs. controls: *p* = 0.05, Figure 7d). Moreover, a slight but insignificant increase in OCR values was detected for *C. parvum*-infected BSIEC in comparison to non-infected controls, (*p* = 0.1) (Figure S3).

## 4. Discussion

In vivo, the life cycle of *C. parvum* occurs in the small intestine—a unique biological niche with a complex, highly structured, and multispecies-composed (i.e., micro- and macrobiota) consortium, which is known to influence intestinal host innate immune reactions, as previously demonstrated [4,44,45,46,47]. This compartment not only promotes nutrient absorption (interestingly, monosaccharide absorption takes place in the ileum, which is also parasitized by *C. parvum*), but also acts as a physical barrier by forming part of the host innate immune system to combat invasive pathogens. These physiological functions are based on a plethora of highly specialized cell types, including intestinal host epithelial cells, which also participate in innate immune responses [13,48]. To mimic this complex system, we used BSI explants cultured under physioxic conditions, which showed a rapid infection-induced switch in metabolic signatures from initially diminished conversion rates of molecules involved in essential metabolic pathways (e.g., glycolysis, amino acid metabolism) at 3 hpi, to reversed conditions with increased metabolic conversion rates at 6 hpi, when compared to respective controls. The parasite-mediated decrease in metabolite conversion rates at 3 hpi may reflect a reduced nutrient uptake by enterocytes—a phenomenon that was previously stated to be a consequence of effective *C. parvum* infection in both human and bovine models [15,49]. Similarly, previous studies reported *C. parvum*-driven pathophysiological changes in the intestine, such as destruction and atrophy of villi and increased epithelial permeability causing diminished nutrient uptake [15,41]—a finding that corresponds well to cases of acute cryptosporidiosis [50,51]. Likewise, we also evidenced intestinal damage by documenting typical hole-like lesions in *C. parvum*-infected villi of BSI explants via SEM analysis. In indirect relation to the increased metabolic conversion rates found in *C. parvum*-infected BSI explants at 6 hpi, Hublin et al. described decreased metabolite content in faecal samples of *C. parvum*-infected mice, which should reflect enhanced metabolite uptake by intestinal epithelial cells [10]. However, direct extrapolation of *C. parvum*-driven metabolic data from murine to human or bovine host systems should be avoided due to host-species-dependent differences, as demonstrated elsewhere [14,49]. Overall, the rapid increase in metabolic conversion rates at 6 hpi being paralleled by a significant increase of *C. parvum* hsp70 gene copies suggests a prompt demand for energy and cell building blocks during early merogony, as also reported in other studies [8,9,13]. Nevertheless, these dynamic metabolic changes need to be further explored using more holistic approaches, thereby considering individual microbiomes, mucus composition, and early intestinal epithelial cell-derived immune reactions against *C. parvum*. Moreover, the present BSI explant-based infection system offers the analysis of bystander cell reactions—such as apoptosis of non-infected epithelial cells; activation of tuft, Paneth, or goblet cells; and leukocyte recruitment—to obtain a more comprehensive overview of total *C. parvum*-driven effects on the intestinal mucosa.

Almost 40 years ago, the first *in vitro* culture system for *Cryptosporidium* was described using human rectal tumour cells (HRT) [52]. This study not only represented a milestone for detailed investigations of cryptosporidiosis *in vitro*, but also elucidated the main handicap of current *Cryptosporidium* culture systems, consisting in the failure to propagate the total parasite life cycle, leading to almost absent gametogony and oocyst production. Meanwhile, a multitude of other host cell types were tested for their suitability as *C. parvum in vitro* infection models. Thus, human-, chicken- and porcine-based cell models—e.g., human foetal lung cells (HFL), primary chicken kidney epithelial cells (PCK), porcine kidney epithelial cells (PK-10) [53], organoids [54,55], COLO-680N cells [56], and air–liquid systems [57]—were described. These all contributed substantially to improvements in *Cryptosporidium* culturing, but were performed under lab-typical, non-physiological hyperoxic (21% O_2_) conditions. Moreover, a systematic implementation and validation of the most successful systems by other research groups seemed limited, based in part on reasons of economic and technical effort, and a lack of reproducibility. Therefore, we here intended to deliver a relatively low-cost, easy, and practicable *in vitro* system to be used in almost all laboratories in industrialized and non-industrialized countries. We here detected glycolytic and glutaminolytic responses early after infection (3 hpi) that signified—to a certain extent—a different scenario than previously reported for *in vitro* experimentation and for murine models [10,12]. However, these data are consistent with *C. parvum*-driven metabolic changes in humans [15]. Thus, the observed reduced glycolytic and glutaminolytic conversion rates at 3 hpi correspond well with higher levels of metabolites (meaning lower metabolic conversion rates) in faecal samples of *C. parvum*-infected humans [15], which could be the result of increased epithelial permeability. Conversely, lower concentrations of the same metabolites (e.g., glucose, glycerol, mannose, alanine, isoleucine, serine, etc.) were found in faecal samples of *C. parvum*-infected mice [10] (pointing at higher metabolic conversion rates), in contrast with the observed reduced metabolic conversion rates of metabolites at 3 hpi. Nevertheless, screening for similarities among metabolic findings from different species or models could be fallacious, since it has been proven that interspecies variation in metabolic profiles of faecal samples is even more extensive than variation due to sample preparation or time [14].

In the present study, we intended to mimic physiological oxygen conditions, which are well documented for their direct effects not only on intestinal key metabolic responses, but also on leukocyte activities [21,49,58,59]. Referring to pyruvate metabolism, *Cryptosporidium*-related anaerobic eukaryotes have been shown to possess oxygen-sensing systems [60,61] coupled to energy metabolism—specifically to enzymes involved in pyruvate degradation. Likewise, *C. parvum* presents a unique pyruvate: NADP+ oxidoreductase partially constituted by a pyruvate:ferredoxin oxidoreductase (PFO) [8,62], which converts pyruvate into acetyl-CoA in final reactions of glycolysis. However, both parasite- and host cell-dependent oxygen-sensing systems may have been involved in increased pyruvate consumption under physioxic conditions at 6 hpi. In mammals, hypoxia-inducible factors (HIFs) represent a well-characterized oxygen-sensing system, regulating a variety of genes in response to low oxygen concentrations [58,61]. Interestingly, HIF-mediated transcription of lactate dehydrogenase genes, leading to enhanced conversion of pyruvate to lactate, may also be linked to increased pyruvate consumption. Serine was found to be produced under hyperoxia but to be consumed under physioxia at 3 hpi. At 6 hpi, serine consumption increased under physioxia and decreased under hyperoxia. Recent findings indicate the relevance of serine as substrate of tryptophan biosynthesis, which is mediated by tryptophan synthase, using serine and indole as substrates [9,63]. Interestingly, both pathogenic species for humans—i.e., *C. parvum* and *C. hominis*—possess a tryptophan synthase-encoding gene (cgd5_4560), which—by contrast—is absent in the closely related species *C. andersoni* [9,64], parasitizing epithelial cells of the stomach, the latter being an endogenous niche characterized by a different pH value and a lack of indole-producing bacteria [65]. Consistently, small intestinal bacteria have been proposed as source of indole for *C. parvum* and *C. hominis*, thereby promoting effective tryptophan biosynthesis [9], which is needed for cell building block-related demands during obligate intracellular parasite replication [13]. However, experimental infections have shown that high faecal indole concentrations (>2.5 nM) were actually associated with protective effects during *Cryptosporidium* infections [17]. It should be noted that indole was also assumed to be a bacterial signalling molecule involved in the regulation of several bacterial parameters, such as virulence, drug resistance, innate immune protection, and biofilm formation [47,66]. Interestingly, low oxygen concentrations promote the growth of distinct bacteria (e.g., γ-Proteobacteria, Vibrionaceae, and Pseudomonadales) [67], thereby influencing indole production [68]. Similarly, it is well known that intestinal microbiomes influence the outcome of several gut infections [44,45,46,47]. This phenomenon was also confirmed for *Cryptosporidium* infections [4,17]. Using the current BSI-explant-based model, we intended to sustain microbiome-driven effects. However, it must be noted that due to experimental procedures during the BSI explant cultures (several washes of explants), only a certain proportion of intestinal bacteria remained in the cultures, since otherwise bacteria would have overgrown too rapidly. Thus, we cannot exclude the possibility that certain microbiomic effects may have been absent. Consequently, future experiments will need to include parallel characterization of individual BSI explant-related microbiomes in order to allow correlations between bacterial species and infection outcome. Interestingly, not only the presence of certain bacteria, but also that of yeast-derived molecules, affects cryptosporidiosis. Similarly, the application of *S. cerevisiae*-based food additives was recently proven as a protective measure in naturally *C. parvum*-infected neonatal calves [4,43]. Even though the related mechanisms are poorly understood, yeast-triggered improvements of gastrointestinal health [69], innate immune responses [70], or development of mucosal resistance towards parasite infection [65,71] have been hypothesized.

In the current study, we additionally analysed BSIEC-related metabolic responses, in order to estimate whether this single cell-type-based primary culture would reflect BSI explant responses. Focusing on glycolysis, we detected infection-triggered changes in BSIEC that in principle paralleled those of BSI explants, since a change from low to high glycolytic responses was detected over time. Interestingly, a parasite-triggered increase in glycolytic activities occurred earlier in BSIEC (at 6 hpi) than in the permanent HCT-8 cell line (at 24 hpi,) [13], which is the most commonly used cell type in *C. parvum*-related *in vitro* studies [13], thereby potentially indicating either a faster parasite development or a more efficient clearance by primary host epithelial cells. As expected, we also detected considerable individual differences in donor-related reactions when using primary bovine epithelial cells. In addition to metabolic responses, infection rates appeared to be highly donor-dependent, and prompted us to separate donors into high and low responders—a finding that well reflects the situation in the field, since the severity of clinical cryptosporidiosis highly varies between calves from the same farm [13,72] or between human patients [73,74].

As previously stated, the impact of microbial consortia in the parasite–epithelial host cell interaction needs to be recognized, and more comprehensive approaches are required in order to address such complex multispecies interactions [22,75].

## 5. Conclusions

The herein-proposed bovine *ex vivo* model offers new useful tools to solve some of the multifactorial questions concerning the triad of *C. parvum*, intestinal epithelial cells, and microbiota. The understanding of such fundamental interactions will improve measures of prevention and unveil new treatment options for cryptosporidiosis in both humans and bovines.

## Data Availability

Not applicable.

## Supplementary Materials

The following are available online https://www.mdpi.com/article/10.3390/biology10100963/s1: Figure S1: *C. parvum* replication up to 48 hpi on BSI explants; Figure S2: Seahorse glycolysis stress test of *C. parvum*-infected BSIEC; Figure S3: *C. parvum*-dependent upregulation of ECAR and OCR in BSIEC at 6 hpi; R_Script for PCA on metabolic data of *C. parvum*-infected and non-infected BSI explants under physioxic (5% O_2_) and hyperoxic (21% O_2_) conditions.
