# Supplementary material for: First Metabolic Insights into Ex Vivo Cryptosporidium parvum-Infected Bovine Small Intestinal Explants Studied under Physioxic Conditions
Source: Biology (Basel). 2021 Sep 26;10(10):963. doi: 10.3390/biology10100963 (PMC8533177; doi:10.3390/biology10100963)
Supplement: Supplementary file 1 [file biology-10-00963-s001.zip › biology-1370745-supplementary.pptx]

## Slide 1
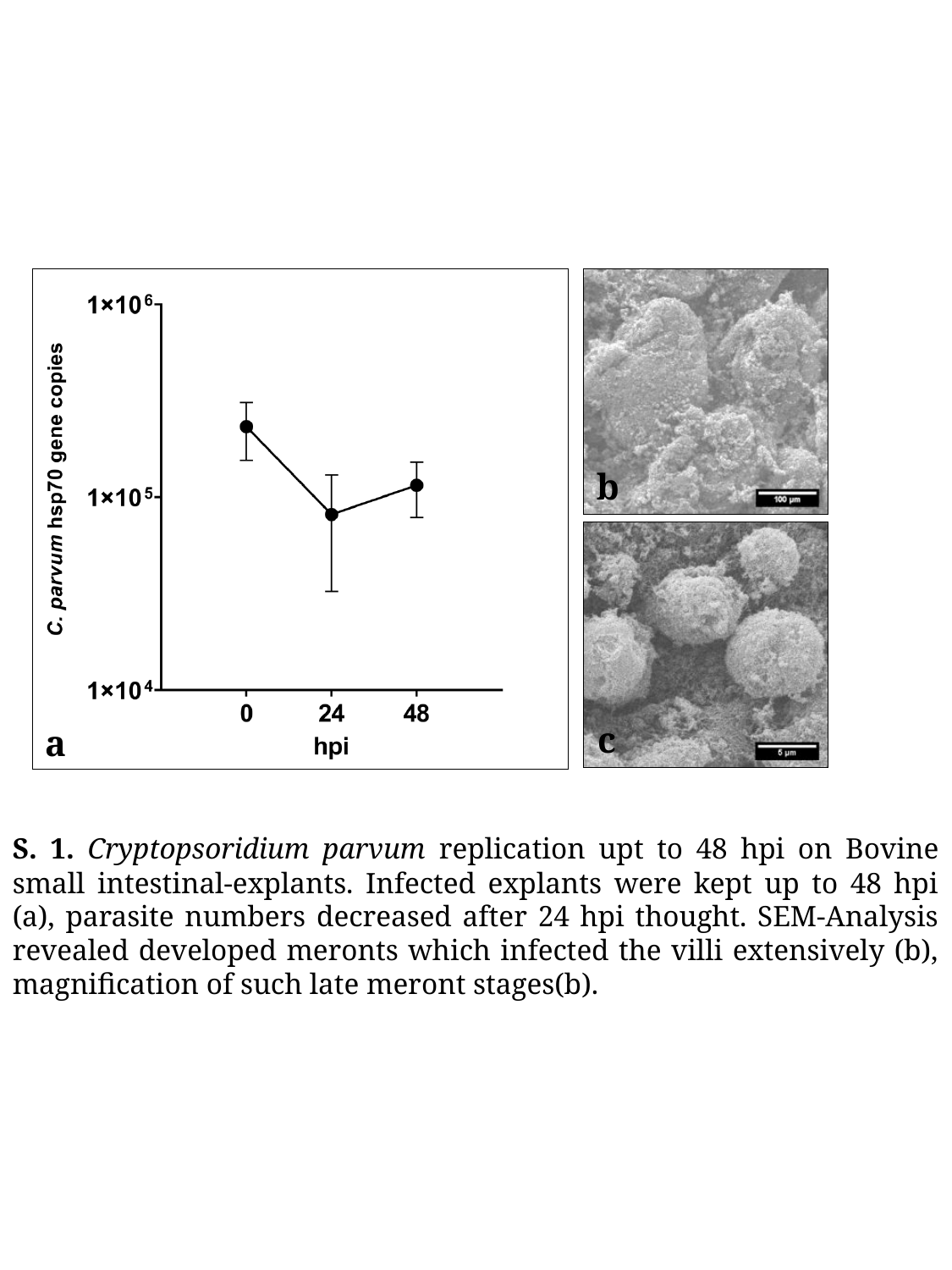

b
c
a
S. 1. Cryptopsoridium parvum replication upt to 48 hpi on Bovine small intestinal-explants. Infected explants were kept up to 48 hpi (a), parasite numbers decreased after 24 hpi thought. SEM-Analysis revealed developed meronts which infected the villi extensively (b), magnification of such late meront stages(b).

## Slide 2
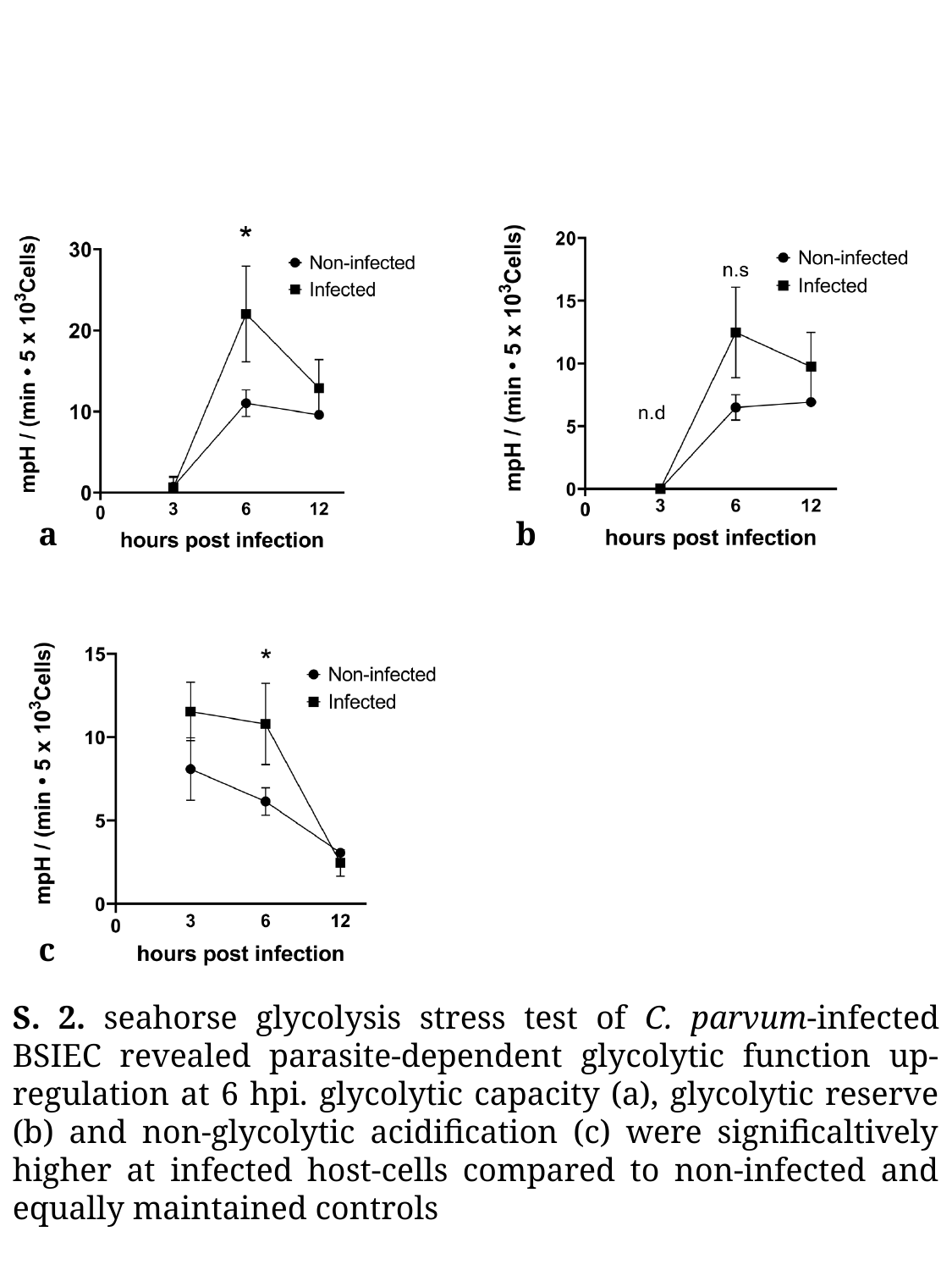

a
b
c
S. 2. seahorse glycolysis stress test of C. parvum-infected BSIEC revealed parasite-dependent glycolytic function up-regulation at 6 hpi. glycolytic capacity (a), glycolytic reserve (b) and non-glycolytic acidification (c) were significaltively higher at infected host-cells compared to non-infected and equally maintained controls

## Slide 3
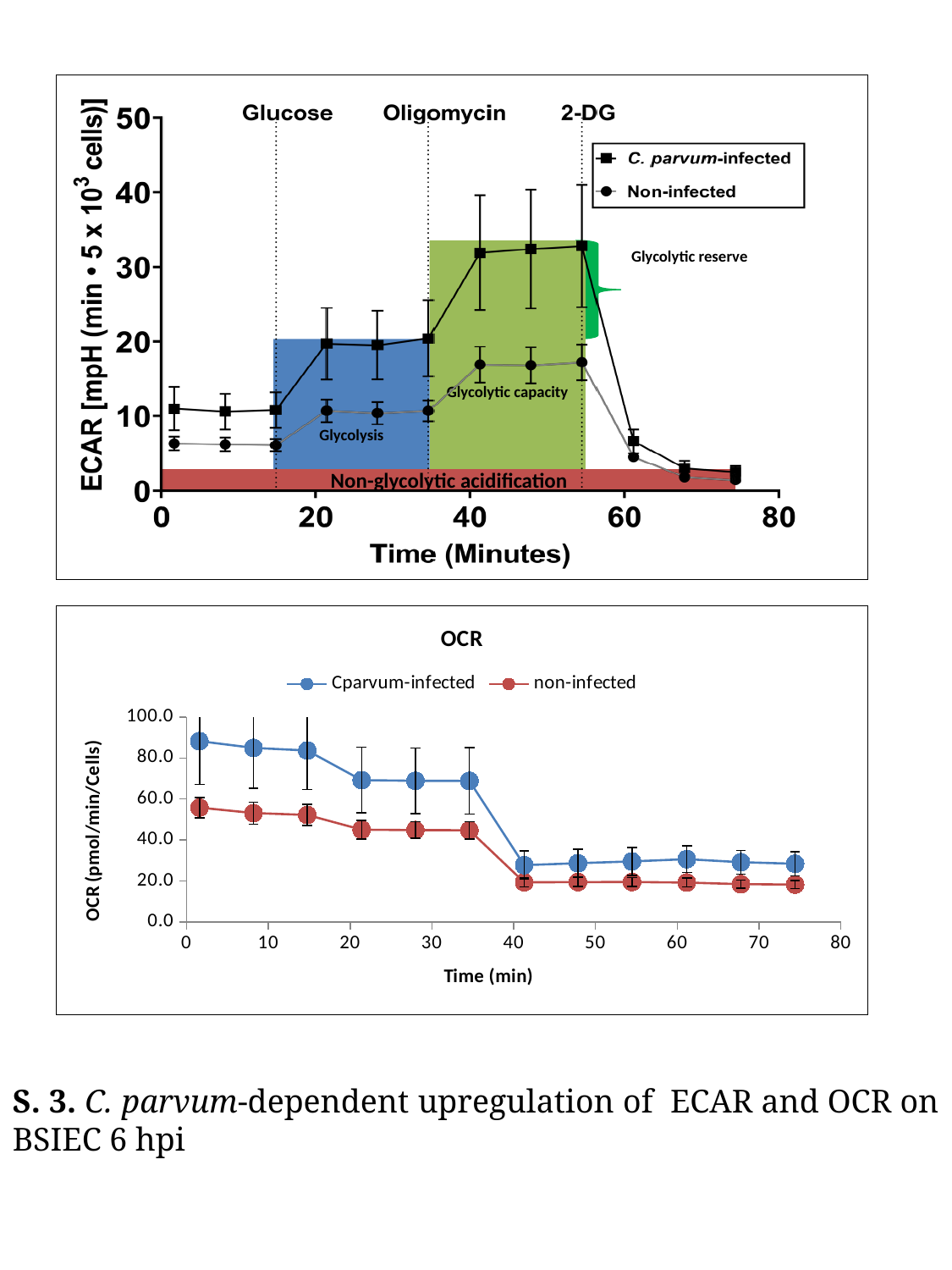

Glycolytic reserve
Glycolytic capacity
Glycolysis
Non-glycolytic acidification
### Chart: OCR
| Category | Cparvum-infected | non-infected |
|---|---|---|S. 3. C. parvum-dependent upregulation of ECAR and OCR on BSIEC 6 hpi
